# Demographics, Socioeconomic Status, Social Distancing, Psychosocial Factors and Psychological Well-Being among Undergraduate Students during the COVID-19 Pandemic

**DOI:** 10.3390/ijerph18147215

**Published:** 2021-07-06

**Authors:** Andréa Neiva da Silva, Carla Ribeiro Guedes, Cláudia Du Bocage Santos-Pinto, Elaine Silva Miranda, Larissa Machado Ferreira, Mario Vianna Vettore

**Affiliations:** 1 Department of Health and Society, Institute of Collective Health, Fluminense Federal University (UFF), Av. Marquês do Paraná, 303/3º andar, Niterói 24070-035, Brazil; andreaneiva@id.uff.br (A.N.d.S.); carlaguedes@id.uff.br (C.R.G.); 2 Integrated Health Institute, Mato Grosso do Sul Federal University (UFMS), Cidade Universitária, Av. Costa e Silva s/n, Campo Grande 79070-900, Brazil; bocage.santos@ufms.br; 3 Departament of Pharmacy and Pharmacy Administration, Faculty of Pharmacy, Fluminense Federal University (UFF), R. Dr. Mario Vianna, 523 Santa Rosa, Niterói 24241-000, Brazil; elainemiranda@id.uff.br (E.S.M.); lamachado@id.uff.br (L.M.F.); 4 Department of Health and Nursing Sciences, University of Agder (UiA), Universitetsveien 25, 4630 Kristiansand, Norway

**Keywords:** COVID-19, undergraduate students, psychological well-being, mental health

## Abstract

The COVID-19 pandemic impacted on academic routine because of the social distancing measures. This study examined the relationships of sociodemographic characteristics, social distancing aspects and psychosocial factors on psychosocial well-being among undergraduate students during the social distancing period due to COVID-19. A web-based survey was conducted of undergraduate students at a public university in Brazil (*n* = 620). Demographics, socioeconomic status (SES), social distancing factors, negative affectivity (DASS-21), sense of coherence (SOC-13), social support and psychosocial well-being (GHQ-12) were measured. The direct and indirect links between was variables was tested using structural equation modelling. The estimated model showed that greater social support, higher sense of coherence and lower negative affectivity were directly associated with better psychological well-being. Female gender, higher SES, not working during the social distancing period and availability of online modules were indirectly associated with psychological well-being through psychosocial factors. Working during the social distancing period and availability of online modules mediated the link of age, gender, SES with psychological well-being. Our findings suggest the need to provide psychological support, online teaching and financial aid to undergraduate students during the social distancing period due to COVID-19 pandemic to improve their psychological well-being.

## 1. Introduction

The new coronavirus disease (COVID-19), initially detected in China in the end of 2020, was quickly disseminated into different countries. Once the SARS-Cov-2 virus related to COVID-19 proved to be highly contagious, the World Health Organization Emergency Committee declared that the outbreak constituted a Public Health Emergency of International Concern [1,2]. The COVID-19 pandemic was considered a global and unprecedented sanitary crisis resulting in the adoption of different measures to reduce the transmission of the disease. Several universities across the world interrupted academic activities. However, in some places, there was a shift from in-campus to online teaching, with educational and mental health implications for nearly 80% of the university students worldwide [3,4]. Of the 69 public federal universities in Brazil, 54 interrupted undergraduate teaching activities during the first semester of 2020 [5].

Previous studies conducted before the COVID-19 pandemic suggested undergraduate students as a vulnerable group of common mental disorders regardless of their country of origin. The symptoms of mental disorders associated with COVID-19 may include insomnia, irritability, fatigue, difficulty concentrating, somatic complaints and forgetfulness [6,7,8]. The prevalence of common mental disorders among university students may range from 19 to 56%, and their occurrence can be influenced by socioeconomic and cultural differences between countries [9,10]. Potential predictors for poor psychological well-being in this group may include female gender [11], lack of social support [12], poor perception of academic environment [13], study a health-related course [14], previous experiences of discrimination [15] and low resilience [16].

University students from different countries remained a vulnerable population group to psychological and health-related problems during the COVID-19 pandemic, including insomnia, anger and fear, alcohol intake and tobacco consumption, anxiety, stress, depression and poor perception of health [17,18,19,20,21].

Therefore, stressful life events, such as the COVID-19 pandemic can directly influence the well-being of individuals through triggering negative affectivity (e.g., anxiety, distress) [22]. Although negative affectivity has been widely investigated due to the potential harmful impact on well-being, research on adaptation processes and how to deal with stressful situations (coping) has gained importance [23,24] as they are central components of the modern theories of psychological well-being. Psychological well-being is a well-established construct in psychological theory involving positive human functioning and optimal development [25]. Positive psychological characteristics are considered protective factors for psychological suffering, and may refer to self-efficacy, and dispositional control [16], self-esteem, positive affection, and resilience [26], vigor [27], extroversion [12] and sense of coherence [9].

Sense of coherence (SOC) consists of a global orientation towards an individual sense of confidence when perceiving life as structured, manageable and coherent. According to Salutogenesis, individuals and social groups may maintain good mental health even when they experience adverse life circumstances [28]. The capacity to withstand threatening situations is possibly related to personal skills and competences as well as the adequate use of available “general resistance resources” to respond to stimuli [29]. These resources may involve financial resources, knowledge, experiences, self-esteem, coping strategies and social support. Social support is an interchangeable system of formal and informal relationships through which people perceive they are cared for, esteemed and belong to a social network of mutual obligations [30]. Social support is characterized by a particular form of collective relationship in which affective exchanges, mutual care and honest and direct communication between people prevail. Social networks are usually established among individuals facing similar daily life circumstances who develop chains of solidary connected with long-lasting patterns of social bonds that occurs in a constant and continuous way [30,31]. Social support has been acknowledged as a meaningful protective factor for psychological well-being among undergraduate students [9].

The adoption of the salutogenic theory in psychological research can enhance the understanding of an individual’s potential resources that facilitate the use of effective coping strategies when experiencing adverse situations. An in-depth assessment of the potential factors related to mental health of university students can contribute to the development of preventive and health promotion strategies to improve their psychological well-being. The aim of this study was to test the relationships between demographic characteristics, socioeconomic status (SES), social distancing aspects, psychosocial factors and psychosocial well-being among undergraduate students during the social distancing period due to COVID-19. It was hypothesized a priori that psychological well-being would be predicted by psychosocial factors, including SOC, social support and negative affectivity, in undergraduate students during the interruption of the academic semester due to COVID-19 pandemic. We also conjectured that the above-mentioned psychosocial factors would mediate the association of demographics, socioeconomic characteristics, and social distancing factors with psychological well-being.

## 2. Materials and Methods

### 2.1. Study Design and Setting

A cross-sectional study using an online web-based survey was conducted of undergraduate students from the Fluminense Federal University, Brazil, during the interruption of the face-to-face teaching activities as a result of the interruption of the academic semester due to COVID-19 pandemic. Fluminense Federal University comprises 60 faculties and institutes grouped into 9 areas, including Exact and Earth sciences, Engineering, Agrarian sciences, Biological Sciences, Health Sciences, Human Sciences, Applied Social Sciences, Linguistics, Language and Arts. There were 45.762 university students enrolled in 131 undergraduate courses. Fluminense Federal University has 3.158 academics and 4.662 administrative staffs distributed across the 10 campuses located in 12 cities in the state of Rio de Janeiro, Brazil. The Niteroi Campus of the Fluminense Federal University is the largest campus involving 24.713 undergraduate students [32].

### 2.2. Participants

University students aged 18 years or older from any gender regularly enrolled in any undergraduate course at the Niteroi Campus of Fluminense Federal University irrespective of year of study were invited to participate. The undergraduate courses at Fluminense Federal University are grouped into three main areas: Life Sciences; Humanities; and Multidisciplinary, Technological and Exact sciences.

Initially, 686 undergraduate students completed the online questionnaire. Of them, 66 were excluded due to missing data in one or more of the following variables: age, gender and reduction of family income during the social distancing period.

The Committee of Ethics and Research of the Fluminense Federal University, Niteroi, RJ, granted ethical clearance for this study (Protocol number 4.132.396). After obtaining consent, the participants completed the online questionnaire.

### 2.3. Sample Size

The minimum sample size was estimated as 538 participants to detect effect size of at least 0.2 (small effect size) in a structural equation modelling involving 4 latent variables and 6 observed variables, considering a significance level of 0.05 and power of 95% [33]. A study with 10% of no acceptance rate or missing data required 600 participants.

### 2.4. Data Collection

A self-completed online questionnaire using Google Forms was used to gather the participants’ data three months after the suspension of the face-to-face teaching activities due to the interruption of the academic semester at Fluminense Federal University as a result of the social distancing measures due to COVID-19 pandemic. Google Forms is a free tool commonly used to collect self-completed questionnaires in online surveys that generates an Excel file with the participants’ responses. Duplicated entry and data manipulation were prevented using the following strategies. First, the Google Form questionnaire was set up to accept only one entry per e-mail address. Second, the online questionnaire could only be accessed once the respondent correctly selected the pictures (e.g., crosswalks) that appeared before the questionnaire. Third, all completed questionnaires were manually checked to avoid duplication using participants’ data of date of birth, university course, height, weight, gender and ethnicity. The present study and the invitation to participate were disseminated to undergraduate students of the university (target population) through different social medias (e.g., Facebook, Instagram and WhatsApp groups). The Students Union social media groups were the starting point of recruitment. Before having access to the online questionnaire, the participants were requested to inform whether they were currently enrolled in any of the undergraduate courses at the Niteroi Campus of the Fluminense Federal University and to inform their institutional email address. Those who did not meet both criteria would be excluded. All respondents fulfilled both criteria and thus no questionnaire was discarded. The recommendations for social distancing in Brazil were announced in the middle of March 2020 and the respondents filled the questionnaire in June 2020.

### 2.5. Measures

#### 2.5.1. Psychological Well-Being

Psychological well-being was a latent variable measured by the 12 items of the Brazilian version of the General Health Questionnaire-12 items (GHQ-12) [34,35]. GHQ-12 is a self-administered screening measure to assess psychological distress in the general population. The GHQ-12 items evaluate whether the respondent has experienced specific symptoms or behaviors related to mental health. The instrument has 12 items and each item is followed by a four-point Likert score. A higher GHQ-12 scores indicates better psychological well-being. The total score of GHQ-12 was calculated by summing the points of the 12 items and may vary from 0 to 36.

#### 2.5.2. Psychosocial Factors

Three psychosocial factors were measured including negative affectivity, sense of coherence (SOC) and social support. The short form of the Depression, Anxiety and Stress Scale-21 items (DASS-21) instrument validated for Brazilian population was used to assess negative affectivity [36,37]. The DASS-21 questionnaire contains 21 items distributed into three subscales (7 items each), corresponding to three dimensions: depression, anxiety and stress. Each item was answered on a four-point Likert scale. Negative affectivity was a latent variable using the scores of each dimension as indicators.

Sense of coherence was assessed according to the Brazilian short version of the Sense of Coherence-13 scale (SOC-13), which consists of 13 items with response options on a five-point Likert scale [28,38]. SOC scores were computed by summing code responses with a final score ranging from 13 to 65. Higher scores of SOC-13 represent stronger levels of SOC [28].

Social support was latent variable measured by the five dimensions of the Brazilian version of the Medical Outcome Study (MOS) social support scale [39,40]. The questionnaire consists of 19 items comprising five dimensions of social support: material (4 items), affective (3 items), emotional (4 items), positive social interaction (4 items) and information (4 items). For each item, the participant is asked to indicate how often they perceive the different types of support using a Likert response scale. A higher social support score indicates stronger perception of social support [40].

All scales used to assess psychological well-being and psychosocial factors in this study were previously validated for the Brazilian population.

#### 2.5.3. Demographics and Socioeconomic Status

Demographics included age and gender (male/female). Socioeconomic status (SES) was a latent variable assessed using three indicators: university admission through social inclusion quotas (yes/no), monthly family income before the social distancing period (up to 3 Brazilian Minimum Wages (BMW), 3–6 BMWs, >6 BMWs), and reduction of family income during the social distancing period (yes/no).

#### 2.5.4. Social Distancing Factors

Social distancing variables were working during the social distancing period (no/yes), availability of online non-mandatory modules during the interruption of the academic semester (no/yes) and whether the student’s city of origin differs from the city of the campus (no/yes).

### 2.6. Data Analysis

Descriptive statistics included means and standard deviation for continuous variables and frequency and percentages for categorical variables for the total sample and according to three groups based on GHQ-12 scores: severe case score category (from 0 to 12), high-risk score category (from 13 to 24), normal state score category (from 25 to 36) [41]. Internal consistencies for the GHQ-12, DASS-21, SOC-13 and the social support scale were evaluated using Cronbach α coefficient.

**Figure 1:**
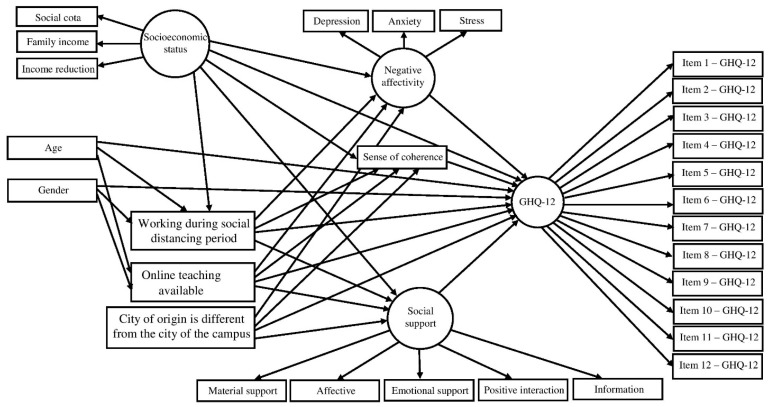
Theoretical framework of the associations between demographics, socioeconomic status, social distancing factors, psychosocial factors and psychological well-being.

Confirmatory factorial analysis (CFA) was used to assess the measurement model and the corresponding indicators of the three latent variables: psychological well-being, socioeconomic status and social support. Direct and indirect associations between variables were tested through structural equation modelling (SEM) according to the proposed theoretical framework (Figure 1). The standardized direct effects represent a direct path from one variable to another, and standardized indirect effects indicate a pathway between two or more variables mediated by another variable. Maximum likelihood method via bias-corrected bootstrap was used to estimate direct and indirect effects and 95% confidence intervals (95% CIs), and to assess whether mediation was present by testing the statistical significance of the indirect effects, with 900 resampling from the original data set in order to derive less biased standard errors [42]. Fit indices were employed to evaluate the adequacy of the measurement and structural models according to the following thresholds: χ^2^/df < 3.0, comparative fit index (CFI) ≥ 0.90, goodness of fit index (GFI) ≥ 0.90 and root-mean-square error of approximation (RMSEA) ≤ 0.06 [43]. The nonsignificant direct paths were removed from the full model to estimate a statistically parsimonious model. Descriptive analysis was performed using IBM SPSS Statistics for Windows, version 21 (IBM Corp., Armonk, NY, USA). CFA and SEM were conducted using SPSS AMOS 24.0 software. The significance level established for all analyses was 5% (*p* ≤ 0.05).

## 3. Results

The studied sample included 620 participants with complete data distributed across the university areas as follows: Life Sciences (51.6%), Humanities (32.7%) and Multidisciplinary, Technological and Exact sciences (15.6%).

The Cronbach’s α coefficient of the GHQ-12 was 0.878, whereas Cronbach’s α coefficients of DASS-21 depression, anxiety and stress scales were 0.908, 0.867 and 0.895, respectively. Cronbach’s α coefficient of the SOC-13 was 0.944, and the Cronbach’s α coefficient of the social support dimensions were: material (α = 0.846), affective (α = 0.880), emotional (α = 0.923), positive social interaction (α = 0.938) and information (α = 0.920).

Demographic characteristics, socioeconomic status, social distancing factors and psychosocial factors are presented for the total sample and according to GHQ-12 groups in Table 1. The average age of the participants was 23.0 (SD = 3.7). The majority of participants were females (78%), admitted through social quotas and from families with monthly income up to 3 BMWs. Most students experienced reduction of family income during the social distancing period, did not work during the social distancing period and were offered non-mandatory online modules during the interruption of the academic semester. The mean scores for psychological well-being, negative affectivity, SOC, and social support were 25.9 (SD = 7.2), 68.2 (SD = 29.8), 37.0 (SD = 8.2) and 57.1 (SD = 17.1), respectively (Table 1).

**Figure 2:**
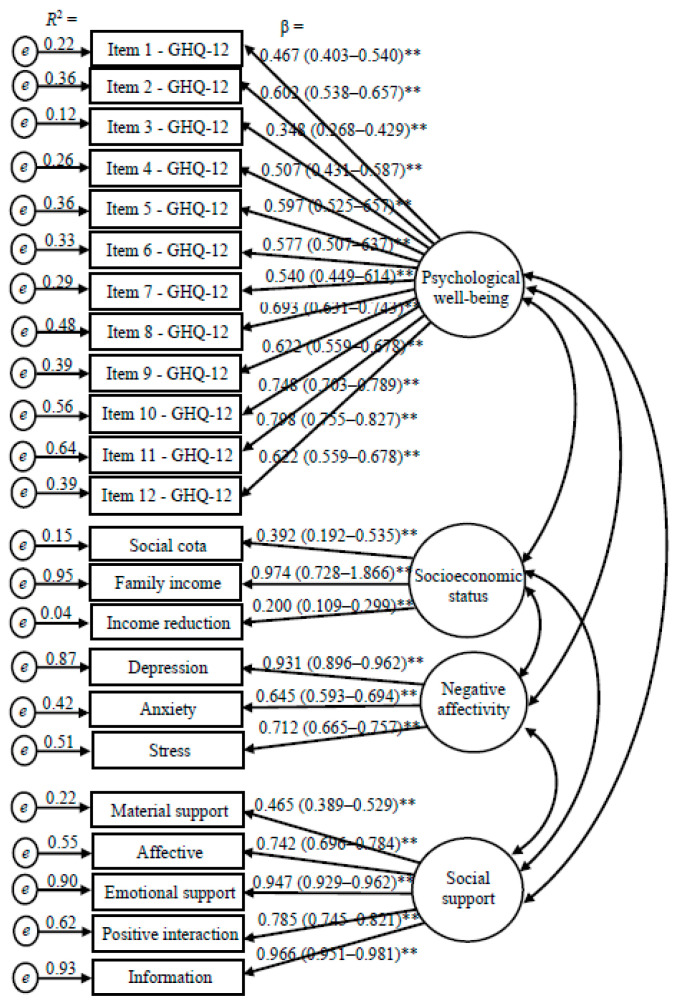
Confirmatory factor analysis of the four-factor and twenty-three-item measurement model obtained through bootstrap item loadings (standard error/bias-corrected 95% CI). ** *p* < 0.01.

The results of the confirmatory factor analysis (CFA) assessing the measurement model for the three latent variables: psychological well-being, socioeconomic status and social support is presented in Figure 2. The latent variable psychological well-being was confirmed according to the 12 items of the GHQ-12 scale. The items that confirmed the latent variable socioeconomic status were admission through social quotas, monthly family income and reduction of family income during the social distancing period. Negative affectivity latent variable was confirmed using the depression, anxiety and stress subscales scores as indicators. Social support latent variable was confirmed using the dimensions of the social support scale as indicators. Measurement model fit indices obtained through CFA were χ^2^/df = 2.5, CFI = 0.959, GFI = 0.924, RMSEA = 0.050. Fit indices of the full model were χ^2^/df = 2.6, CFI = 0.938, GFI = 0.904, RMSEA = 0.051. Structural equation modelling supported the parsimonious model with the following fit indices: χ^2^/df = 2.6, CFI = 0.940, GFI = 0.905, RMSEA = 0.051.

**Figure 3:**
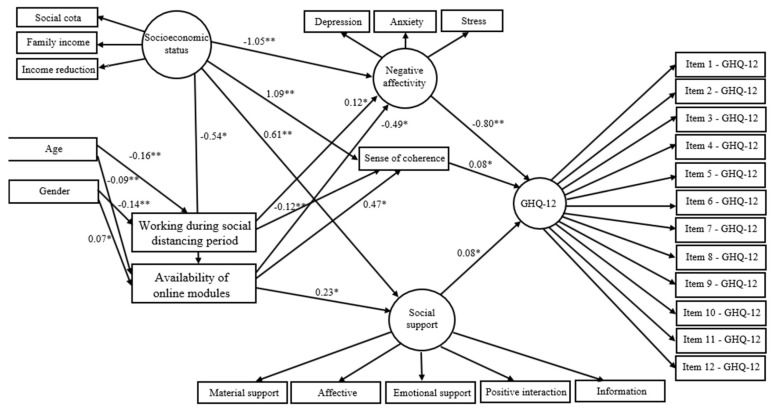
Direct effects showing the standardized estimates for the parsimonious model. * *p* < 0.05, ** *p* < 0.01.

The variable student’s city of origin differs from the city of the campus was removed due to lack of significant association with other variables in the full model. Non-significant direct paths were also removed to obtain the statistical parsimonious. The direct relationships of the parsimonious model are summarized in Figure 3. Lower negative affectivity (β = −0.80), higher SOC (β = 0.08), greater social support (β = 0.08) were directly related to better psychological well-being. Better socioeconomic status directly predicted lower negative affectivity (β = −1.05), higher SOC (β = 1.09) and greater social support (β = 0.61). Lower age was directly linked to working during the social distancing period (β = −0.16) and no availability of online modules (β = −0.09). Female gender directly predicted not working during the social distancing period (β = −0.14) and availability of online modules (β = 0.07). Working during the social distancing period was directly related to higher negative affectivity (β = 0.12) and lower SOC (β = −0.12). Availability of online modules was directly associated with lower negative affectivity (β = −0.49), higher SOC (β = 0.47) and higher social support (β = 0.23).

Significant indirect relationships of socioeconomic status (β = 0.73), gender (β = 0.05), working during the social distancing period (β = −0.10) and availability of online modules (β = 0.45) with psychological well-being were identified. Socioeconomic status indirectly predicted negative affectivity (β = 0.27) and SOC (β = −0.25) via availability of online modules. Female gender was indirectly related to negative affectivity (β = −0.05) and SOC (β = 0.05). The parameters of the direct and indirect effects are described in Table 2.

## 4. Discussion

The main finding of this study is that better psychological well-being among undergraduate students was related to protective psychosocial factors, including higher sense of coherence and greater social support, during the period of interruption of the academic semester due to the COVID-19 pandemic. Negative affectivity, which is a multidimensional construct involving depression, anxiety and stress, was also linked to worse student’s psychological well-being. Moreover, the above-mentioned psychosocial factors mediated the links of demographic and socioeconomic characteristics, working during the social distancing period and availability of online modules during the interruption of the academic semester with psychological well-being.

Our findings support the direct effect of psychosocial factors on psychological well-being among university students during the social distancing period related to interruption of the academic semester due to COVID-19. Recent studies have revealed the impact of depression, anxiety and stress on mental health of medical students and adolescents during the COVID-19 pandemic [44,45]. SOC was considered an important predictor of psychological well-being among youths [46], representing a source of individual protection when facing stressful situations [47]. Therefore, the participants of the present study with higher SOC might understand the COVID-19 pandemic as comprehensible, manageable and meaningful situation. Possibly, they were also able to mobilize a number of resources and develop skills to deal with the adverse scenario related to the pandemic context, which in turn resulted in better psychological well-being.

Similar to our findings, previous research has shown the increased risk of mental health problems among university students with low social support during the new coronavirus pandemic [44,48,49,50]. Feelings of loneliness and poor perception of family environment resulted in psychological distress during the pandemic [49]. Living alone, lack of social interaction with relatives and close friends and weak social ties with other students are the main explanations for the harmful effects of low social support on poor mental health [51]. Thus, social support is a protective factor for psychological well-being among undergraduate students during the pandemic. Social support is a psychosocial coping resource that can attenuate the negative effects of stress [52] and positively influence the emotional health of the individuals [53,54], especially during strict social distancing times. Social support also results in mutual benefits to the members of social groups, helping them to cope with daily challenges and contributing to the maintenance of their physical and psychological health [30,31].

Our study demonstrated the mediating effect of negative affectivity, SOC and social support on the relationship between socioeconomic status and psychological well-being. There is sound evidence on the mediating effect of psychosocial factors on the above-mentioned relationship [55]. The mediation of adolescent’s SOC on mental health problems during difficult situations has been reported [56]. It is noteworthy to mention that the development and structuring process of SOC occur during adolescence and early adulthood [47]. So, a higher family socioeconomic status can contribute to a greater development of SOC during adolescence and thus reduce psychological suffering among youths [57]. On the other hand, the relationship of low income and lower social support with mental problems among university students has been shown [58].

In this study, negative affectivity and SOC mediated the relationship between working during the social distancing period due to pandemic and psychological well-being. Working during the pandemic possibly increased anxiety and stress among students due to a higher perception of exposure to the virus. The impact of paid work on student’s mental health was reported [59]. However, working during the COVID-19 pandemic negatively affected student’s SOC, which in turn influenced their psychological well-being [47]. It has been shown that SOC can be restructured as an aftermath of traumatic and unexpected events such as the pandemic [28,60].

The present findings suggest the availability of online modules during the interruption of the academic semester indirectly influenced the psychological well-being through psychosocial factors. Remote online teaching can be considered a source of stress among university students during pandemic [44,61]. For instance, difficulties in adapting to online teaching was associated with psychological distress among medical students [44]. However, our study indicated the offer of online teaching during the social distancing period contributed to the reduction of student’s psychological suffering. Attending online courses probably strengthened their sense of belonging, favoring their capacity to face difficulties and contributing to their psychological well-being. The reduced offer of online courses among those with high family income contributed to worst psychological well-being through psychosocial factors. Therefore, offering online courses during the COVID-19 pandemic was very important for student’s mental health regardless of their socioeconomic status.

Working during the interruption of the academic semester due to the COVID-19 pandemic had a negative impact on psychological well-being among the youngest participants. This group was predominantly composed by adolescents, a period of life characterized by physical, emotional and social changes that increases their vulnerability to mental health problems [62]. Nonetheless, a greater offer of online modules during the social distancing period positively influenced psychological well-being, mainly among the younger students via psychosocial factors. The mediating effect of working during the pandemic and lower availability of online modules during the interruption of the academic semester on psychological well-being were noticeable among female students. Previous research reported that female students experienced worse mental health than male students during the COVID-19 pandemic in different countries [43,51]. Moreover, working or living with someone working during social distancing was related to lower levels of psychological distress among undergraduate students [44].

Institutional and governmental initiatives may improve student’s psychological well-being during the period of interruption of the academic semester due to the COVID-19 pandemic. Overall, in this study nearly 40% of the students were from low-income families and 61% of the participants reported reduction of family income during the social distancing period. Therefore, the provision of financial aid and scholarships to vulnerable students in order to prevent these students working during the COVID-19 pandemic may reduce negative effects and consequently enhance their psychological well-being.

Whilst poor physical health may have been implicated with distress as a potential source of anxiety and stress, health status measures were not assessed in this study. The reason for not investigating participant’s physical health was essentially because undergraduate students are mostly composed of younger adults who are in good physical health. Therefore, physical health was out of the scope of the study.

The shortcomings of this study should be acknowledged and the results must be interpreted with caution. The data was collected using an online questionnaire. Thus, the degree of randomness of the sample is difficult to verify. Even though, the number of participants was higher than the pre-established sample size, students without internet access, mainly those of low-income background, were unable to participate. Furthermore, students from health and biological sciences courses were overrepresented in the studied sample (51.6%), which proportion is much higher than the total of students enrolled in those courses (19.3%) [32]. There was also a higher proportion of female participants (78% of the sample). So, our findings should be interpreted with caution due to the above-mentioned sampling issues. These figures might explain the high proportion of severe cases of poor psychological well-being (low scores of GHQ-12). Previous studies revealed the relationship of university students enrolled in health subjects [63,64] and female students with greater psychological distress [11]. Data collection was conducted four months after the interruption of the academic semester without any expected date of return. This blurring scenario may have contributed to the higher levels of psychological stress among the participants. Furthermore, this was a cross-sectional study and as a consequence, causal–effect relationships between variables must not be implied.

The psychosocial impacts related to the mitigation measures to reduce the transmission of the COVID-19 infection may impact on people’s mental health. Thus, psychosocial support measures have been recommended to tackle mental health problems during social distancing periods [65]. The findings of this study suggest the need for planning and delivering remote psychological care services for undergraduate students, such as psychosocial support and support groups, in order to reduce negative affectivity, reinforce SOC and enhance social support, especially during the interruption of the academic calendar due to the COVID-19 pandemic. Timely psychological support can prevent worsening the psychological disorders in general population as well as among students during the pandemic [65]. Previous studies have shown the effect of psychological interventions on increasing individual’s SOC that might be relevant among young undergraduate students when their SOC are challenged by the pandemic [66,67]. A recent systematic review reported that digital interventions directed to mental health problems were effective to reduce depression and anxiety, and to improve psychological well-being among university students [68].

Our data also indicate the importance of offering remote online modules by universities to enhance student’s psychological well-being when academic calendar is suspended. Universities should provide electronic devices as well as internet access to students, mainly for those living in social vulnerability.

Brazil is struggling to control the COVID-19 infection as the number of new cases and deaths reached a high and stable plateau since January 2021. In addition, the delay in vaccination and the lack of effective physical measures (e.g., lock down) combined with insufficient social protective measures suggest an unfavorable scenario in the forthcoming months. The lack of foresight to return to classrooms in the universities presents itself as an important challenge for student’s psychological well-being, mainly among those from lower socioeconomic status, younger students, and females. These groups should receive psychological and financial support during the social distancing periods related to the COVID-19 pandemic.

## 5. Conclusions

Our study demonstrated that SOC and social support were meaningful factors associated with better psychological well-being among under-graduate students during the period of interruption of the academic semester at the university due to the COVID-19 pandemic. Moreover, negative affectivity had a negative impact on student’s psychological well-being. Thus, the hypothesis that psychosocial factors would predict student’s psychological well-being during the interruption of the academic semester due to COVID-19 pandemic was confirmed.

The second hypothesis of this study was also accepted since working during the social distancing period, age, and socio-economic status were indirectly associated with psychological well-being through psychosocial factors.

## Figures and Tables

**Table 1:** Socio-demographics, factors related to disruption of face-to-face classes, and psychosocial factors of the participants: comparison between psychological well-being groups.

Variables	Total (*n* = 620)	Psychological Well-Being		
		Severe Case Score Category (*n* = 279/45%)	High-Risk Score Category (*n* = 287/46.3%)	Normal State Score Category (*n* = 54/8.7%)
	Mean (SD)	Mean (SD)	Mean (SD)	Mean (SD)
Age, mean (SD)	23.0 (3.7)	23.2 (4.1)	22.8 (3.5)	22.8 (2.2)
	*N* (%)	*N* (%)	*N* (%)	*N* (%)
Gender				
Female	484 (78)	229 (82)	222 (77)	33 (61)
Male	136 (22)	50 (18)	65 (23)	21 (39)
Admission through social quotas				
Yes	262 (42)	124 (44)	115 (40)	23 (43)
No	358 (58)	155 (56)	172 (60)	31 (57)
Monthly Family income				
Up to 3 BMWs	236 (38)	118 (42)	101 (35)	17 (32)
3–6 BMWs	194 (31)	98 (35)	77 (27)	19 (31)
>6 BMWs	190 (31)	63 (23)	109 (38)	18 (33)
Reduction of family income during the social distancing period				
Yes	377 (61)	180 (65)	168 (59)	29 (54)
No	243 (39)	99 (36)	119 (42)	25 (46)
Working during the social distancing period				
No	496 (80)	231 (83)	227 (79)	38 (70)
Yes	124 (20)	48 (17)	60 (21)	16 (30)
Availability of online modules				
No	279 (45)	141 (50)	119 (42)	19 (35)
Yes	341 (55)	138 (50)	168 (59)	35 (65)
City of origin is different from the city of the campus				
No	353 (57)	156 (56)	167 (58)	30 (56)
Yes	267 (43)	123 (45)	120 (42)	24 (44)
	Mean (SD)	Mean (SD)	Mean (SD)	Mean (SD)
Negative affectivity	34.1 (14.9)	44.6 (10.2)	27.7 (11.8)	13.9 (8.9)
Depression	11.5 (5.8)	15.8 (4.0)	8.8 (4.4)	3.9 (3.5)
Anxiety	9.0 (5.8)	12.3 (5.1)	7.0 (5.0)	3.2 (3.5)
Stress	13.5 (5.2)	16.6 (3.8)	11.9 (4.7)	6.8 (4.2)
SOC	37.0 (8.2)	31.5 (6.4)	40.4 (6.1)	47.1 (6.2)
Social support	57.1 (17.1)	50.5 (17.8)	61.2 (14.5)	69.2 (9.4)
Material support	13.3 (3.6)	12.6 (4.1)	13.8 (3.0)	14.5 (3.2)
Affective support	9.7 (3.0)	8.7 (3.4)	10.3 (2.5)	11.3 (1.5)
Emotional support	14.3 (2.8)	9.2 (4.7)	12.0 (4.1)	14.3 (2.8)
Positive social interaction	11.9 (4.6)	10.3 (4.7)	12.8 (3.9)	14.7 (2.6)
Information support	11.3 (4.4)	9.6 (4.5)	12.3 (3.9)	14.6 (2.1)

BMW: Brazilian Minimum Wage.

**Table 2:** Direct and indirect effects of the parsimonious structural equation model.

Pathways	β	95% CI	*p*
Direct Effects			
Negative affectivity → psychological well-being	−0.80	−0.92/−0.68	0.003 **
SOC → psychological well-being	0.08	0.05/0.20	0.016 *
Social support → psychological well-being	0.08	0.02/0.14	0.014 *
SES → Negative affectivity	−1.05	−2.00/−0.87	0.002 **
Working during the social distancing period → Negative affectivity	0.12	0.03/0.19	0.013 *
Availability of online teaching → Negative affectivity	−0.49	−1.72/−0.03	0.038 *
SES → SOC	1.09	0.93/2.08	0.001 **
Working during the social distancing period → SOC	−0.12	−0.19/−0.05	0.005 **
Availability of online teaching → SOC	0.47	0.01/1.74	0.040 *
SES → social support	0.61	0.49/1.02	0.002 **
Availability of online teaching → social support	0.23	0.04/0.87	0.009 **
Age → working during the social distancing period	−0.16	−0.26/−0.07	0.002 **
Gender → working during the social distancing period	−0.14	−0.23/−0.05	0.002 **
SES → availability of online teaching	−0.54	−0.90/−0.13	0.019 *
Age → availability of online teaching	−0.09	−0.16/−0.03	0.004 **
Gender → availability of online teaching	0.07	0.01/0.14	0.032 *
Indirect effects			
SES → psychological well-being	0.73	4.05/27.29	0.001 **
Gender → psychological well-being	0.05	0.01/0.16	0.001 **
Working during the social distancing period → psychological well-being	−0.10	−0.21/−0.04	0.01 *
Availability of online teaching → psychological well-being	0.45	0.02/1.56	0.042 *
SES → Negative affectivity	0.27	1.22/4.51	0.018 *
Gender → Negative affectivity	−0.05	−2.68/−0.22	0.001 **
SES → SOC	−0.25	−4.61/−0.43	0.036 *
Gender → SOC	0.05	0.32/2.86	0.001 **

* *p* < 0.05; ** *p* < 0.01.

## Data Availability

The data that support the finding of this study can be available on request from the corresponding author following a one-year embargo from the date of publication. The data are not publicly available due to sensitive information that could compromise the privacy of research participants.
